# Study protocol for the PICASSO trial: A randomized placebo-controlled trial to investigate the efficacy and safety of intraarticular steroid injections and an occupational therapy intervention in painful inflammatory carpometacarpal-1 osteoarthritis

**DOI:** 10.1016/j.ocarto.2024.100542

**Published:** 2024-11-19

**Authors:** Marthe Gløersen, Ingvild Kjeken, A.T. Tveter, Amirhossein Kazemi, Joseph Sexton, Krysia Dziedzic, David T. Felson, Tanja A. Stamm, Ali Guermazi, Merete Hermann-Eriksen, M.I. Sæther, Kristine Lundby, E.L. Esperø, Monika Olsen, K.B. Norheim, Edle Berg Fister, Mari Hoff, Jorunn Kvalø Uleberg, Irina Petrovna Midtgard, Therese Andreassen, Dag Sjølie, Heidi Sletten, H.B. Hammer, Ida K. Haugen, the PICASSO study team, Åshild Hove, Alexander Mathiessen, Lena Bugge Nordberg, Even Lillejordet, Adrian Gran, Åse Klokkeide, Maia Muri Aursand, Sofie Ryvoll Åsheim, Anne Lillerud Slagsvold, Shagaye Nabizadeh, Göran Karlsson, Thalita Blanck, Sissel Bærø Nyheim, Trine Amalie Sjøvold

**Affiliations:** q Center for Treatment of Rheumatic and Musculoskeletal Diseases (REMEDY), Diakonhjemmet Hospital, Oslo, Norway; r Martina Hansens Hospital, Bærum, Norway; s Haugesund Rheumatism Hospital, Haugesund, Norway; t St. Olav's Hospital, Trondheim University Hospital, Trondheim, Norway; u Nordland Hospital, Bodø, Norway; v Norwegian Rheumatism Association, Norway; a Center for Treatment of Rheumatic and Musculoskeletal Diseases (REMEDY), Diakonhjemmet Hospital, Oslo, Norway; b Oslo Metropolitan University, Oslo, Norway; c Institute for Primary Care and Health Sciences, Arthritis Research UK Primary Care Centre, Keele University, Keele, UK; d Rheumatology Section, Chobanian & Avedisian Boston University School of Medicine, Boston, MA, USA; e Medical University of Vienna, Center for Medical Data Science, Institute for Outcomes Research, Vienna, Austria; f Ludwig Boltzmann Institute for Arthritis and Rehabilitation, Vienna, Austria; g Department of Radiology, Chobanian & Avedisian Boston University School of Medicine, Boston, MA, USA; h Martina Hansens Hospital, Bærum, Norway; i Haugesund Rheumatism Hospital, Haugesund, Norway; j Stavanger University Hospital, Stavanger, Norway; k University of Bergen, Faculty of Medicine, Bergen, Norway; l St. Olav's Hospital, Trondheim University Hospital, Trondheim, Norway; m Department of Neuromedicine and Movement Science, Norwegian University of Science and Technology, Trondheim, Norway; n Nordland Hospital, Bodø, Norway; o Department of Radiology, Diakonhjemmet Hospital, Oslo, Norway; p University of Oslo, Faculty of Medicine, Oslo, Norway

**Keywords:** Osteoarthritis, Hand osteoarthritis, Pain, Clinical trial

## Abstract

**Objective:**

Our primary objectives are to assess whether intraarticular corticosteroid injections are superior to saline injections with regards to thumb base pain after 4 weeks, and to compare the efficacy of steroid injections, saline injections, and an occupational therapy intervention on thumb base pain after 12 weeks in people with painful inflammatory osteoarthritis (OA) of the first carpometacarpal (CMC-1) joint.

**Design:**

In this three-armed, double-blind, randomized multicenter trial, 354 participants with painful inflammatory CMC-1 OA from six Norwegian hospitals are recruited. Participants are randomized 1:1:1 to intraarticular steroid or saline injections in the CMC-1 joint or a multimodal occupational therapy intervention. The primary outcomes are thumb base pain measured on a numeric rating scale (NRS, range: 0–10) after 4 weeks and 12 weeks. Key secondary outcomes include synovitis by Magnetic Resonance Imaging (MRI) after 4 weeks and hand function by the Measure of Activity Performance of the Hand (MAP-Hand) questionnaire after 12 and 24 weeks. Other secondary outcomes are synovitis by clinical examination and ultrasound, measures of pain, function, stiffness, and health-related quality of life, and direct and indirect costs. Adverse events are recorded at each visit. The duration of the randomized controlled trial is 24 weeks, followed by an 80-week open-label observational phase to investigate the long-term efficacy and safety of repeated steroid injections and the occupational therapy intervention.

**Conclusions:**

The results from this trial will have important clinical implications and influence future guidelines on OA management of the CMC-1 joint.

**Clinical trial registration:**

EU-CT 2023-505254-17-00, NCT06084364.

## 1 Introduction

Osteoarthritis (OA) of the first carpometacarpal (CMC-1) joint is a common condition, which may lead to pain and reduced function [1]. Synovitis is often present in CMC-1 OA and may represent a treatment target due to its associations with symptoms and OA progression [[2], [3], [4], [5]]. Intraarticular corticosteroid injections are frequently used to treat synovitis in osteoarthritic CMC-1 joints in clinical practice, but previous studies have not shown a clinically beneficial effect on pain in the CMC-1 joint compared with saline or local anesthesia injections [[6], [7], [8]]. However, previous studies published in full-text were small with only approximately 20 participants per treatment arm, and did not require participants to have inflammation in the CMC-1 joint at inclusion [6,7]. One larger study published as an abstract presented data after 6 months only [8].

International hand OA guidelines propose evaluating the efficacy of steroid injections in osteoarthritic CMC-1 joints with inflammation as a research priority [9]. A recent study demonstrated that 10 ​mg (mg) of oral prednisolone daily for 6 weeks had a clinically relevant effect on pain compared with placebo in patients with hand OA, and a previous study of intraarticular steroid injections in the interphalangeal joints showed an effect on pain during movement [10,11]. In knee OA, a meta-analysis found that steroid injections were more effective than placebo on pain in the short term (≤6 weeks) [12]. We hypothesize that intraarticular steroid injections may have a beneficial short-term effect on pain also in persons with painful inflammatory CMC-1 OA.

International guidelines state that a multimodal occupational therapy intervention that consists of education, hand exercises, use of assistive devices and training in ergonomic principles should be offered to all patients with hand OA [9]. A mobile self-management application can facilitate successful delivery of information about OA, training in ergonomic principles and hand exercises [13]. Multimodal occupational or physical therapy interventions have demonstrated beneficial effects on pain in thumb base OA in two systematic reviews [14,15]. A recent randomized controlled trial of a multimodal occupational therapy intervention demonstrated a significantly better effect on pain, function and grip strength after 3 months compared with information about hand OA only [16]. The long-term effect of multimodal occupational therapy has not been comprehensively studied, and no previous studies have compared intraarticular steroid injections with a multimodal occupational therapy intervention in CMC-1 OA.

### 1.1 Project aims and objectives

The main aim of the PICASSO trial is to assess the efficacy and safety of intraarticular steroid injections and a multimodal occupational therapy intervention in people with CMC-1 joint OA. We will also explore the long-term efficacy of the interventions and safety of repeated steroid injections in an open-label extension phase.

## 2 Method

### 2.1 Study design and setting

**Fig. 1:**
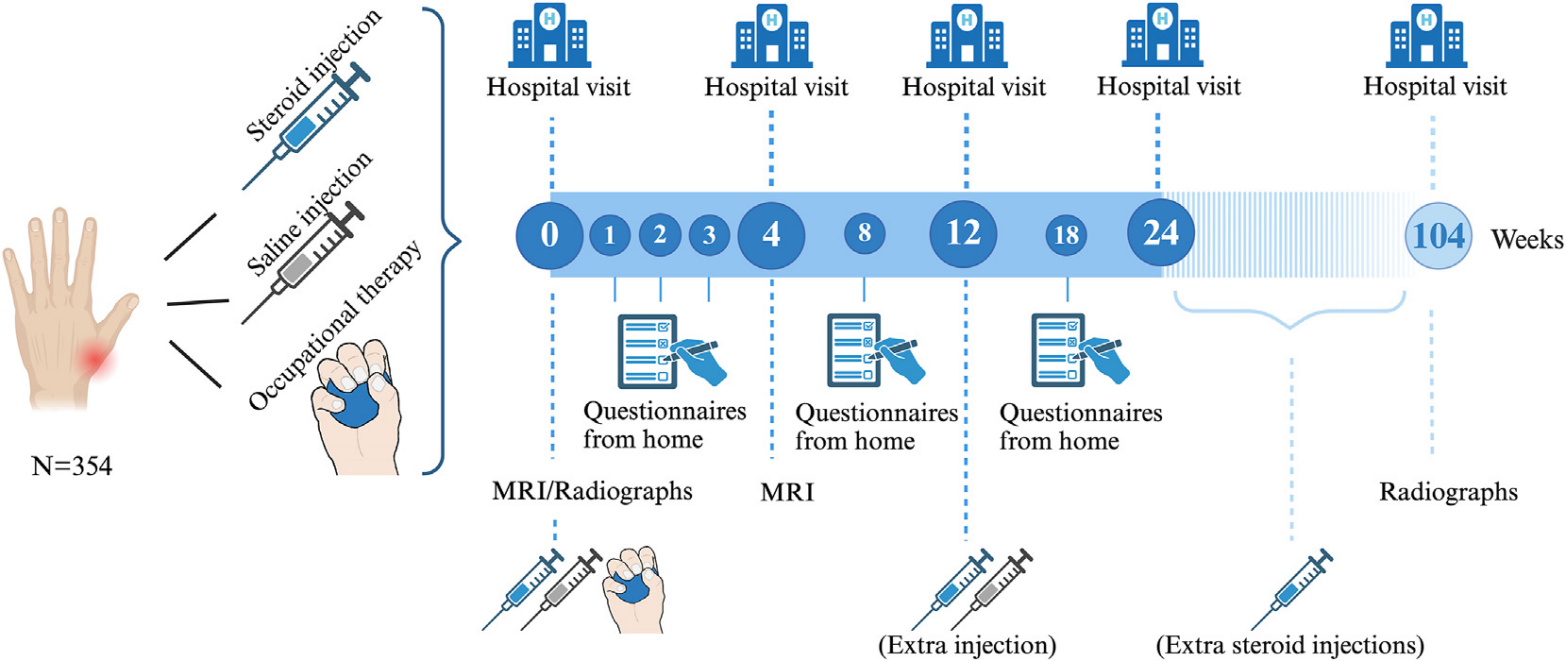
PICASSO timeline and overview of the interventions. Created with BioRender.com. MRI ​= ​magnetic resonance imaging.

The Painful Inflammatory Carpometacarpal-1 osteoArthritis treated with intraarticular Steroids, Saline or an Occupational therapy intervention (PICASSO) trial is a three-armed, investigator-initiated, double-blind randomized controlled multicenter trial with a duration of 24 weeks (Phase 1), followed by an 80-week open-label observational phase (Phase 2), in which all participants may receive intraarticular steroid injections or other treatments if indicated. Participants are randomized to intraarticular steroid injections, intraarticular saline injections, or a multimodal occupational therapy intervention. Fig. 1 illustrates the study design. Participants are recruited from six Norwegian hospitals (Diakonhjemmet Hospital, Martina Hansens Hospital, Haugesund Rheumatism Hospital, Stavanger University Hospital, St. Olav's University Hospital and Nordland Hospital Bodø). The data collection is ongoing. The first study participant completed the baseline visit in December 2023, and we anticipate that recruitment will take approximately 2 years.

### 2.2 Participants

Participants with painful inflammatory CMC-1 OA and no evidence of systemic inflammatory joint diseases, psoriasis, fibromyalgia, or serious comorbidities are included in the trial. Table 1 describes inclusion and exclusion criteria. Participants are mainly recruited through referrals to rheumatology or orthopedic surgery outpatient clinics at the hospitals or through advertisements in media.

**Table 1:** Inclusion and exclusion criteria in the PICASSO trial.

**Inclusion criteria**
• Adults between 40 and 85 years of age In target CMC-1 joint^a^: • OA by radiographs (description of osteophytes, joint space narrowing or degenerative changes in previous radiographs) or ultrasound (osteophyte grade ≥1 on a 0–3 scale) • Ultrasound-defined synovitis (grey-scale synovitis grade ≥1 on a 0–3 scale) • Pain at rest or during activities (grade ≥3 on a 0–10 scale)^b^
**Exclusion criteria**
• Thumb orthosis or structured hand exercises on most days during the last 12 weeks • Intraarticular injections in target CMC-1 joint during the last 12 weeks • More than 3 previous steroid injections in target CMC-1 joint • Use of oral, intravenous or intramuscular steroids during the last 12 weeks • Previous surgery of target CMC-1 joint • Planned hand surgery during the next 24 weeks • Not willing to stop using oral or topical NSAIDs during the next 12 weeks^c^ • A systemic inflammatory joint disease or other disease that can explain hand pain • Diagnosis of fibromyalgia • Diagnosis of psoriasis • Skin disease, infection or wound at the target CMC-1 joint • Serious comorbidities, cognitive dysfunction, substance or alcohol abuse or other conditions that make it difficult to follow the study protocol • Severe or uncontrolled infection • Known hypersensitivity to triamcinolone acetonide (Kenacort-T) or the excipients • Participation in other clinical studies • Use of digitalis glycosides • Vaccination with live virus vaccines last 2 weeks • Not able to understand or speak Norwegian • Planned pregnancy during the next 24 weeks or known pregnancy • Any other condition that suggests that the patient cannot comply with the study protocol or procedures

CMC-1 ​= ​first carpometacarpal; NSAIDs ​= ​non-steroidal anti-inflammatory drugs; OA ​= ​osteoarthritis.

a The following rule is applied to select a target CMC-1 joint for all participants in the trial: The CMC-1 joint with ≤3 previous intraarticular steroid injections, no injections in the last 12 weeks and without previous surgery should be selected if participants have two painful inflammatory CMC-1 joints with OA. If these exclusion criteria are not present in any of the CMC-1 joints, the joint with the most severe pain should be chosen as the target joint. If both CMC-1 joints are equally painful, the joint with the most severe grey-scale synovitis should be chosen. The dominant hand should be chosen if still equal. Only this target joint is treated with intraarticular injections or orthosis (in the occupational therapy group) during the first 24 weeks of the trial. After 24 weeks, all participants may receive steroid injections and both hands may be treated.

b Pain should be ​≥ ​3 on a 0–10 scale at both pre-screening and screening.

c Oral NSAIDs and use of topical NSAIDs on the hands are not allowed in the first three months of the trial where the primary outcomes are measured, unless they are used as rescue medication (i.e. up to 3 days per week if adequate pain control is not achieved with basic pain medication such as paracetamol alone or in combination with a low-dose codeine, in agreement with the study personnel). After 12 weeks, NSAIDs are allowed, but preferably not the last 48 ​h before a study visit.

### 2.3 Patient and public involvement

Three patient research partners (TB, SBN, TAS) with hand OA have been involved in planning the study design, giving input on the assessments and questionnaires, the informed consent form and information letters. They will also be consulted during the conduction of the trial, and in discussions and dissemination of results.

### 2.4 Endpoints

The primary endpoints are thumb base pain during activities in the last 24 ​h after 4 and 12 weeks assessed on 0–10 Numeric Rating Scales (NRS). Key secondary endpoints include synovitis on MRI after 4 weeks and hand function by the Measure of Activity Performance of the Hand (MAP-Hand) questionnaire after 12 and 24 weeks. Several secondary endpoints are measured, including pain, function, stiffness, grip strength, quality of life, inflammation, adverse events, and direct and indirect costs (Table 2). Adverse events are recorded at each visit.

**Table 2:** Overview of measurements.

Measurements	Time point (weeks)^a^
**Primary endpoints**	
• Thumb base pain during activities (NRS)	• 4, 12
**Secondary endpoints**	
• Thumb base pain during activities (NRS) • Thumb base pain at rest (NRS) • Finger joint pain (NRS) • Disease activity (NRS) • AUSCAN hand pain, stiffness and function • MAP-hand • OMERACT/OARSI responder criteria • EQ-5D-5L • Pain in finger joints (hand figure) • Self-efficacy (ASES) • Questions related to health economics^b^ • Life cycle analyses^c^ • Adverse events and safety • Tender and swollen hand joint counts • Assessor-reported disease activity • Grip strength • Bilateral hand radiographs • MRI of the target CMC-1 joint • Ultrasound of the CMC-1 joint • Use of analgesics and NSAIDs	• 0, 1, 2, 3, 8, 18, 24, INJ, 104 • 0, 4, 12, 24, 104 • 0, 4, 12, 24, 104 • 0, 4, 12, 24, 104 • 0, 4, 12, 24, 104 • 0, 4, 12, 24, 104 • 0, 4, 12, 24, 104 • 0, 4, 12, 24, 104 • 0, 4, 12, 24, 104 • 0, 4, 12, 24, 104 • 0, 12, 24, 104 • 24, 104 • 0, 4, 12, 24, INJ, 104 • 0, 4, 12, 24, 104 • 0, 4, 12, 24, 104 • 0, 4, 12, 24, 104 • 0, 104 • 0, 4 • 0, 4, 12, 24, INJ, 104 • 0, 1, 2, 3, 4, 8, 12, 18, 24, 104
**Tertiary/exploratory endpoints**	
• Question about blinding • Question about experienced effect • Kapandji index • Flexion deficit 2nd-5th finger	• 4, 12 • 4, 12, 24 • 0, 4, 12, 24, 104 • 0, 4, 12, 24, 104
**Demographics and clinical characteristics**	
• Demographics, life style factors, symptom duration, previous treatments, preferred treatment, pain in other joints, anxiety/depression (HADS), pain catastrophizing • Comorbidities • Medication list • Use of corticosteroids • Use of hand exercises, thumb orthosis, aids and the happy hands app • Quality indicator set for hand OA • American College of Rheumatology and EULAR classification criteria for hand OA • Height and weight	• 0 • 0, 4, 12, 24, INJ, 104 • 0, 4, 12, 24, INJ, 104 • 0, 4, 12, 24, 104 • 24, 104 • 0, 12, 24 • 0 • 0

ACR= American College of Rheumatology; AUSCAN=Australian/Canadian Osteoarthritis Hand index; ASES= Arthritis self-efficacy scale; CMC-1 ​= ​first carpometacarpal; EQ-5D-5L ​= ​The EuroQol 5 dimensions 5 levels; EULAR=European Alliance of Associations for Rheumatology; HADS=Hospital Anxiety and Depression Scale; INJ ​= ​visit with steroid injection in Phase 2;MAP-Hand ​= ​Measure of Activity Performance of the Hand; MRI ​= ​Magnetic Resonance Imaging; NRS=Numeric rating scale; NSAIDs ​= ​Non-steroidal anti-inflammatory drugs; OA=Osteoarthritis; OARSI=Osteoarthritis Research Society International; OMERACT=Outcome Measures in Rheumatology.

a Week 0 includes both assessments at screening and at baseline.

b Health economics: A cost-utility analysis of steroid injections and occupational therapy, where direct and indirect healthcare costs are calculated, will be performed. We will collect data about intervention-related resource use, healthcare service use, and labor market status. The EuroQol 5 dimensions 5 levels (EQ-5D-5L) questionnaire will be used to collect descriptions of the participants´ health-related quality of life.

c Life cycle analyses: The carbon footprint of intraarticular injections and occupational therapy will be assessed by life cycle analyses. Data about for example medications administered, medical equipment used, use of electrical energy, transport, waste, and number and types of consultations will be converted into CO2 equivalents.

### 2.5 Randomization and blinding

Participants are allocated in a 1:1:1 ratio to receive intraarticular steroid injections in CMC-1, intraarticular saline injections in CMC-1 or a multimodal occupational therapy intervention. A central computer randomization procedure stratified by study center is used, and the randomization list is integrated into the electronic case report form Viedoc (Viedoc™, Uppsala, Sweden).

**Fig. 2:**
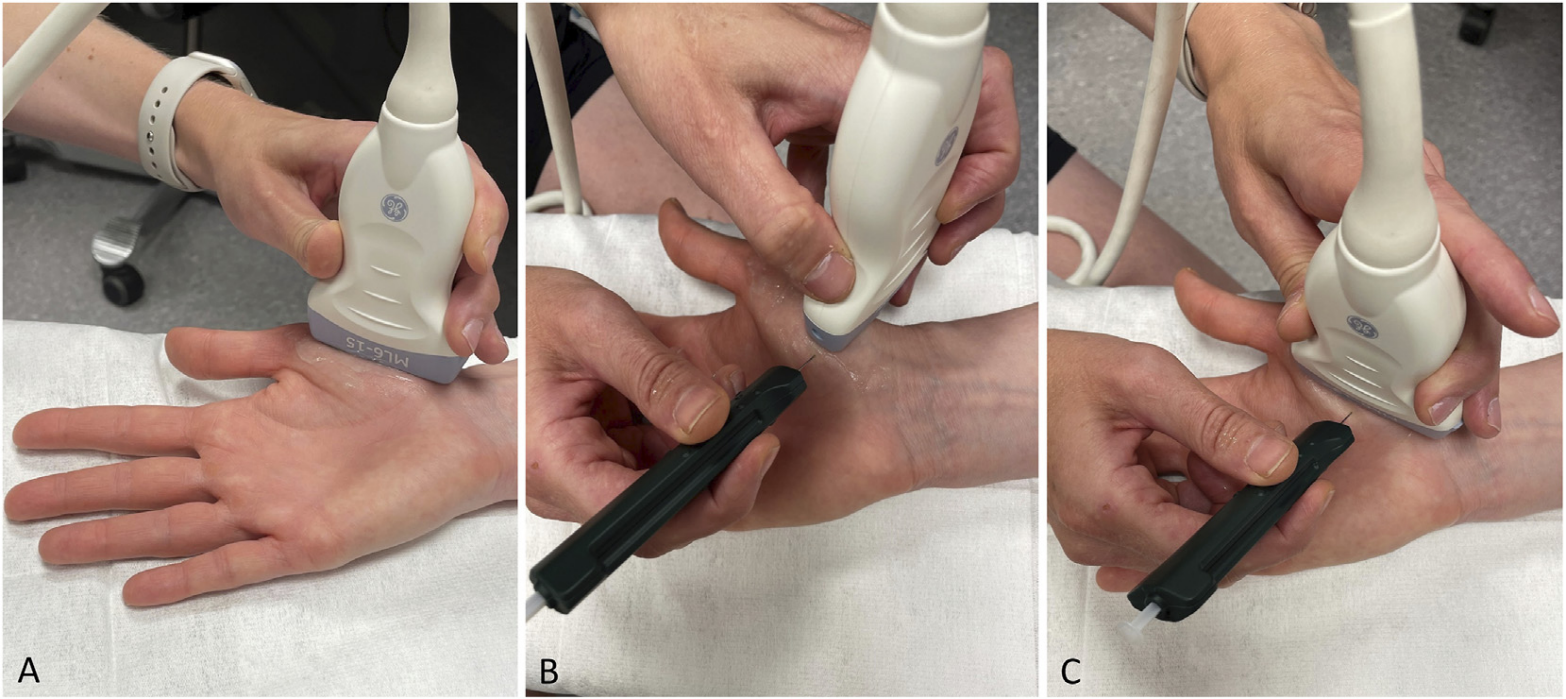
Intraarticular ultrasound-guided injection in the CMC-1 joint.

The occupational therapists are informed whether participants are randomized to an injection or the occupational therapy intervention. Both participants and occupational therapists are blinded to syringe content (i.e. steroid vs. saline). The trained rheumatologists or rheumatology fellows who perform the injections are not blinded to content since it is possible to differentiate steroids from saline on the ultrasound image. This is unlikely to influence the study results, since all examinations are performed by an occupational therapist, and the rheumatologists or rheumatology fellows are not involved in these examinations. A non-transparent syringe shield (Syrigma™) covers the syringe to ensure blinding of the participants (Fig. 2). The multimodal occupational therapy intervention cannot be performed with blinding. To minimize the contextual response, the study personnel deliver neutral information about the interventions.

### 2.6 Procedures

#### 2.6.1 Intraarticular injections in the CMC-1 joint

At baseline, a syringe covered with an opaque syringe shield is filled with 0.5 ​mL (ml) triamcinolone acetonide (Kenacort-T 40 ​mg/ml) or 0.9 ​% sterile saline (Sodium chloride Fresenius Kabi 9 ​mg/ml) in a separate room or concealed from the participant. An ultrasound-guided injection is performed by a rheumatologist or trained rheumatology fellow. Prior to the study, all rheumatologists and rheumatology fellows involved in the study get access to a video showing the injection procedure, which can be accessed throughout the study if needed: https://www.youtube.com/watch?v=sB5p9YRX7FM. The injection is given under aseptic conditions with disinfection of the injection site and non-touch technique. The joint space of the CMC-1 joint is first identified in the longitudinal plane (Fig. 2, picture A). The probe is then turned 90° and the needle is inserted into the CMC-1 joint in transverse scan (Fig. 2, picture B). To confirm that the needle is placed correctly, the probe may be turned back in longitudinal plane (Fig. 2, picture C). When correct placement is confirmed, 0.5 ​ml (20 ​mg) of triamcinolone acetonide or 0.5 ​ml 0.9 ​% sterile saline is injected. Ultrasound is used throughout the procedure to ensure correct intraarticular placement of the needle. A smaller dosage may be given if increased resistance develops during the injection procedure, and the volume injected is noted. After the injection, the site is covered and a pressure is applied to the injection site. The participant is advised to rest the hand for 24 ​h. An injection with the same content (triamcinolone acetonide or saline) as at baseline is given at the 12-week visit if there is still pain (≥3 on a 0–10 NRS) and inflammation (grey-scale synovitis grade 1–3) in the CMC-1 joint.

#### 2.6.2 Multimodal occupational therapy intervention

The intervention consists of patient education, information about assistive devices and ergonomic working methods, and hand exercises, initiated in a face-to-face consultation, and thereafter supported by a self-management application called Happy Hands [13]. When indicated, participants also receive a day and/or night thumb orthosis for the target CMC-1 joint. Orthosis design is tailored to the degree of CMC-1 OA, deformities, and subluxation. The Happy Hands package is a 12-week intervention with informational videos and a hand exercise program. The 25 informational videos address the following themes: Information about hand OA, motivation and self-efficacy, assistive devices and ergonomic principles, hand exercises, orthoses, and communication, and are given in a progressive order for 12 weeks. The exercise program has increasing intensity over 12 weeks, and consists of 8 exercise videos. These include a warm-up exercise, exercises to improve range of motion and grip strength, exercises to strengthen the 1st dorsal interossei and the stability of the wrist and arm muscles, exercises to improve proprioception and neuromuscular control of the CMC-1 joint and a stretching exercise. All participants receive equipment to perform the exercises, including a rubber ball for grip strength training, rubber band for arm strength training and tennis ball for training of proprioception, and are encouraged to exercise 3 times per week. They rate their pain and stiffness after each exercise session, and their development over the 12 weeks is visualized on graphs based on their answers. The application also provides weekly quizzes, encouragement, and motivational messages.

### 2.7 Assessments

Candidates undergo a pre-screening assessment by phone, where most inclusion and exclusion criteria are assessed. Potentially eligible participants attend a screening visit at the hospital, where an occupational therapist obtains informed consent before they assess all inclusion and exclusion criteria. The baseline visit, where participants are randomized to one of the interventions, is scheduled within 3 weeks after screening. MRI of the target CMC-1 joint is obtained between screening and baseline, and is repeated after 4 weeks. Radiographs of the bilateral hands are obtained at baseline and the 2-year visit. Participants attend hospital visits after 4, 12 and 24 weeks, after 2 years, and in case of repeated injections in Phase 2. A timeline is included in Fig. 1.

#### 2.7.1 Questionnaires

Participants answer self-reported questionnaires at hospital visits, and some questions at home after 1, 2, 3, 8 and 18 weeks (Table 2). Most participants answer the questionnaires electronically in Viedoc, but the questionnaires are available in paper form if needed.

#### 2.7.2 Clinical examination

Clinical examination is performed by an occupational therapist and includes a hand joint examination of soft tissue swelling and joint tenderness according to the European Alliance of Associations for Rheumatology (EULAR) handbook, verification of the American College of Rheumatology and the EULAR classification criteria, measurement of Kapandji Index and flexion deficit of the second to fifth fingers, height and weight, grip strength by a Jamar dynamometer, and registration of comorbidities, medications and previous treatment (Table 2) [[17], [18], [19], [20], [21], [22]]. Adverse events are assessed at every hospital visit.

#### 2.7.3 Imaging

Bilateral hand radiographs (front images) are obtained in posteroanterior view at baseline (±3 weeks) and after 2 years (±3 weeks). The source to image-receptor distance is 115 ​cm, exposure 46 ​kVp and 2 ​mAs. Validated scoring systems are used by a trained central reader to score OA pathology in the hand joints, including the CMC-1 joint [[23], [24], [25]].

**Fig. 3:**
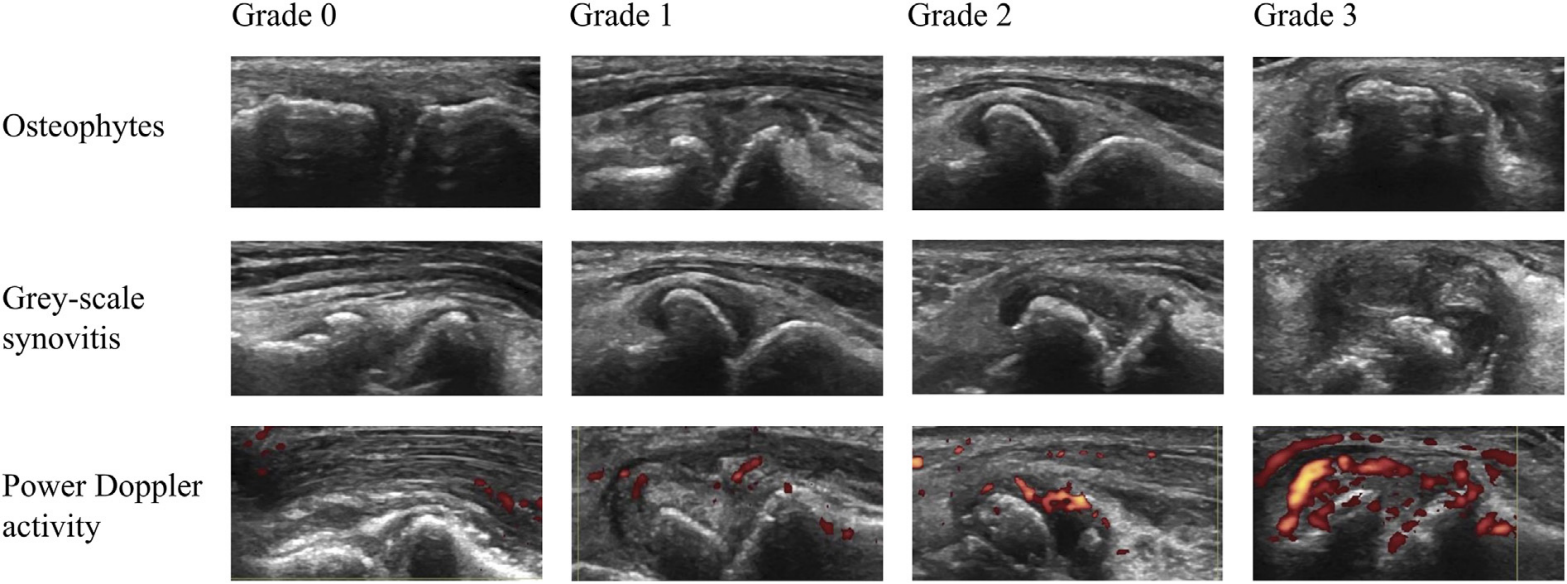
Example images from the ultrasound atlas of CMC-1 joints with grade 0 (no pathology), grade 1 (mild), grade 2 (moderate) and grade 3 (severe) pathology.

Ultrasound is performed by occupational therapists who have received training in ultrasonography. Longitudinal scanning of the bilateral CMC-1 joints is performed at the volar side from radial to ulnar side, and a transverse scan is performed if there is uncertainty about pathology. Osteophytes and grey-scale synovitis are assessed to investigate the inclusion criteria at screening, whereas grey-scale synovitis and power Doppler activity are scored on 0–3 scales at each hospital visit [26]. We have developed an atlas based on atlases that were previously developed by members of our research group [27,28]. This atlas is used during scoring to increase reliability. Example images from this atlas are shown in Fig. 3. A reliability exercise between the occupational therapists and an experienced ultrasonographer was performed in the beginning of the study with good reliability between each occupational therapist and an expert ultrasonographer (weighted kappa ≥0.60) [29].

MRI of the target CMC-1 joint is performed by a minimum of 1.5T whole-body MRI scanner with a dedicated hand coil. MRI is performed between screening and baseline and±7 days from the hospital visit after 4 weeks. Intravenous gadolinium contrast (Clariscan 0.5 ​mmol/mL, 0.2 ​mL/kg body weight) is used. MRI will not be obtained in participants with an estimated glomerular filtration rate ​< ​40 ​mL/min or with other contraindications to intravenous contrast or MRI scanning in itself. Creatinine and estimated glomerular filtration rate are measured before MRI is obtained at baseline. MRI is performed with participants lying in a supine position with their feet first and their hand with the target CMC-1 joint along their side. Details of the sequences are described in Table 3. A trained central reader will score the MRIs for synovitis and bone marrow lesions by using validated scoring systems [30].

**Table 3:** Details of the MRI sequences.

	Coronal PD Dixon	Transversal PD Dixon	Coronal T1 VIBE Pre contrast	Coronal T1 VIBE Post contrast	Total scan time
Slice thickness (mm)	2	3	0,3	0,3	
Spacing (mm)	0,6	0,6	0	0	
Pixel size	0,3i	0,2i	0,2i	0,25i	
Time (min:sec)	2:20	3:06	3:15	3:15	11:56

PD ​= ​proton density, i ​= ​interpolated, VIBE ​= ​volumetric interpolated breath-hold examination.

### 2.8 Statistical analyses

Descriptive statistics will be used to summarize demographics and baseline characteristics, primarily on the intention-to-treat population. Our primary analysis will be carried out in the modified intention-to-treat population of randomized persons receiving interventions. Analyses will be repeated for the per-protocol population. The pair-wise comparison between intraarticular steroid and saline injections after 4 weeks will be performed by analysis of covariance, adjusting for baseline NRS pain and study center. Hierarchical testing will be employed using an F-test to compare the intraarticular steroid injections, saline injections, and occupational therapy group at 12 weeks. If the F-test shows a significant between-group difference, pair-wise comparisons will be performed in the same way as at 4 weeks. To control for multiplicity, the week 4 test will be performed at the 0.025 level. If steroid injections are significantly superior to saline injections with a significance level of <0.025 ​at ​week 4, the week 12 test will be performed at 0.05 level. If steroid injections are not significantly superior to saline at week 4 (p ​≥ ​0.025), the week 12 test will be performed at 0.025 level.

We will perform secondary subgroup analyses of participants with grey-scale and MRI-defined synovitis grade <2 versus ≥2 (0–3 scale), with and without subluxation of the CMC-1 joint, with and without power Doppler activity in the target thumb base joint and of participants with pain 0-2 versus 3–10 in the contralateral thumb base joint. Results from the first 24 weeks of the trial will be analyzed when the last participant has completed the 24-week visit.

We performed a simulation study to determine the sample size. To detect an NRS pain difference of 1.2 (0–10 scale), we required 80 ​% conjunctive power (probability of rejecting all null hypotheses given that none of them are true). This is based on results from the HOPE trial, which showed a difference in thumb base pain of 12 on a 0–100 Visual Analogue Scale in participants receiving oral prednisolone versus placebo [10]. We assume a standard deviation of 2.5 points on the NRS scale, which is based on results from three studies (the HOPE trial, a trial of intraarticular steroid injections, and a trial of a multimodal occupational therapy intervention) [8,10,16]. Based on the simulation, 319 participants are needed. We will recruit 354 participants to account for 10 ​% loss to follow-up.

### 2.9 Ethics and dissemination

Informed consent is obtained from all participants before they are included in the trial. Participants can withdraw from the trial at any time without providing a reason. The PICASSO trial is registered at https://clinicaltrials.gov (NCT06084364). The Norwegian Regional Committee for Medical and Health Research Ethics and the Norwegian Medical Products Agency has approved the study through the clinical trials information system (EU CT number: 2023-505254-17-00). Data protection officers at the involved hospitals have approved the trial. The procedures are in accordance with the Helsinki Declaration.

The main ethical issue in the trial is possible side effects of intraarticular steroid injections, but these are usually unserious or rare. Both intraarticular steroid and saline injections pose a risk of bleeding or infection, although this risk is low. There is an interval of at least three months between the injections, which is in line with common clinical practice.

Data is collected in a web-based electronic case report form software solution (Viedoc™, Uppsala, Sweden). Data from Happy Hands is stored in Services for Sensitive Data (TSD), University of Oslo. The document with the link between the participant identification number in Viedoc and name is stored securely in a locked cabinet at each hospital.

Results from the trial will be submitted to international peer-reviewed journals, and uploaded to the Clinical Trials Information System within the first year after the end of the study. Results will also be disseminated to study participants.

## 3 Discussion

The PICASSO trial is the first large multicenter trial to assess the efficacy and safety of intraarticular steroid injections in painful inflammatory CMC-1 joint OA, and to compare the efficacy of steroid injections with a multimodal occupational therapy intervention. Previous trials of steroid injections in CMC-1 joint OA have failed to demonstrate superior effect of steroids on pain compared with saline or local anesthesia injections [[6], [7], [8]]. Our study may be less prone to false negative results due to inclusion of patients with grey-scale synovitis on ultrasound and pain, appropriate dosage of steroids, ultrasound-guided injections and larger sample size. Our primary endpoints are pain after 4 weeks to compare the efficacy of intraarticular steroid and saline injections, and pain after 12 weeks to compare injections with the occupational therapy intervention. The reason for comparing the efficacy of steroid versus saline injections after 4 weeks is based on a meta-analysis showing a beneficial effect of steroid injections compared with placebo on short-term pain (≤6 weeks) in people with knee OA, and a hand OA study that demonstrated a clinically relevant effect of prednisolone after 6 weeks [10,12]. The effect of the occupational therapy intervention is expected to take longer to materialize, and we chose to assess the effect after 12 weeks since the Happy Hands intervention continues for 12 weeks and adherence may decrease thereafter.

We included several secondary and exploratory endpoints, including various measures of pain, function, stiffness, disease activity, self-efficacy, health-related quality of life, patient satisfaction, grip strength, tender and swollen joint counts, use of healthcare services, adverse events, ultrasound- and MRI-detected synovitis, MRI-detected bone marrow lesions and structural progression on radiographs. Due to this broad data collection, several research questions will be explored to gain new knowledge about CMC-1 OA. We will explore the long-term effects and safety of repeated steroid injections and of multimodal occupational therapy in a two-year follow-up period.

It has previously been shown that the size of the contextual response is largest when the placebo is delivered through injections [31], which may impact the results. On the other hand, one can argue that participants in the occupational therapy arm are aware that they get an active intervention due to lack of blinding, in contrast to those who receive injections. To minimize the contextual response, study personnel are instructed to provide neutral information about the interventions.

The results from this trial may directly improve clinical practice, and influence guidelines for treatment of CMC-1 joint OA.

## Author contributions

All co-authors had substantial contributions to the conception or design of the work, or the acquisition, analysis or interpretation of data. MG drafted the paper. All coauthors revised it critically for important intellectual content and gave their final approval of the version published. MG and IKH take responsibility for the integrity of the work as a whole, from inception to finished article.

## Role of the funding source

The PICASSO trial is funded by the Norwegian National Program for Clinical Treatment Research in the Specialist Health Service (KLINBEFORSK). The REMEDY center was funded by The Research Council of Norway (project number: 328657). The funders were not involved in the study design, data collection, writing the manuscript or the decision to submit the manuscript.

## Declaration of competing interest

**AG** reports consulting fees from Pfizer, TissueGene, Formation Bio, ICM, Coval, Novartis and Medipost, and stock/stock options in BICL and LLC, outside of the submitted work. **KD** is part funded by the National Institute for Health and Care Research (NIHR) Applied Health Research Collaboration (ARC) West Midlands (NIHR 200165) and the Birmingham Biomedical Research Centre (BRC). KD is also an NIHR Senior Investigator (ID NIHR 205031). The views expressed are those of the author(s) and not necessarily those of the NIHR or the Department of Health and Social Care. **HBH** reports honorarium from Abbvie, Novartis, Lily and UCB, and advisory boards for Abbvie and Novartis, outside of the submitted work. **HS** reports patent issued and pending for Syrigma™ (syringe cover). **IKH** reports personal fees from Novartis, GSK and Grünenthal, and speaker honorarium from Abbvie, outside of the submitted work. **TAS** reports grants and personal fees from Roche, and personal fees from AbbVie, Roche, Sanofi, Takeda, and Novartis, outside the submitted work. **AK, ATT, DTF, DS, EBF, ELE, IK, IPM, JKU, JS, KBH, KL, MG, MH, MHE, MIS, MO** and **TA** report no conflicts of interest.

## References

[1] Haugen I.K., Englund M., Aliabadi P., Niu J., Clancy M., Kvien T.K., Felson D.T. (2011). Prevalence, incidence and progression of hand osteoarthritis in the general population: the Framingham Osteoarthritis Study. Ann. Rheum. Dis..

[2] Fjellstad C.M., Mathiessen A., Slatkowsky-Christensen B., Kvien T.K., Hammer H.B., Haugen I.K. (2020). Associations between ultrasound-detected synovitis, pain, and function in interphalangeal and thumb base osteoarthritis: data from the nor-hand cohort. Arthritis Care Res..

[3] Kortekaas M.C., Kwok W.Y., Reijnierse M., Watt I., Huizinga T.W., Kloppenburg M. (2010). Pain in hand osteoarthritis is associated with inflammation: the value of ultrasound. Ann. Rheum. Dis..

[4] Mathiessen A., Slatkowsky-Christensen B., Kvien T.K., Hammer H.B., Haugen I.K. (2016). Ultrasound-detected inflammation predicts radiographic progression in hand osteoarthritis after 5 years. Ann. Rheum. Dis..

[5] Haugen I.K., Slatkowsky-Christensen B., Boyesen P., Sesseng S., van der Heijde D., Kvien T.K. (2016). MRI findings predict radiographic progression and development of erosions in hand osteoarthritis. Ann. Rheum. Dis..

[6] Heyworth B.E., Lee J.H., Kim P.D., Lipton C.B., Strauch R.J., Rosenwasser M.P. (2008). Hylan versus corticosteroid versus placebo for treatment of basal joint arthritis: a prospective, randomized, double-blinded clinical trial. J Hand Surg Am.

[7] Meenagh G.K., Patton J., Kynes C., Wright G.D. (2004). A randomised controlled trial of intra-articular corticosteroid injection of the carpometacarpal joint of the thumb in osteoarthritis. Ann. Rheum. Dis..

[8] Mandl L.A., Wolfe S., Daluiski A., Hotchkiss R.N., Lyman S.L., Katz J.N. (2012). A randomized controlled trial of hylan G-F 20 for the treatment of carpometacarpal osteoarthritis (abstract). Arthritis Rheum..

[9] Kloppenburg M., Kroon F.P., Blanco F.J., Doherty M., Dziedzic K.S., Greibrokk E. (2019). 2018 update of the EULAR recommendations for the management of hand osteoarthritis. Ann. Rheum. Dis..

[10] Kroon F.P.B., Kortekaas M.C., Boonen A., Böhringer S., Reijnierse M., Rosendaal F.R. (2019). Results of a 6-week treatment with 10 mg prednisolone in patients with hand osteoarthritis (HOPE): a double-blind, randomised, placebo-controlled trial. Lancet.

[11] Spolidoro Paschoal Nde O., Natour J., Machado F.S., de Oliveira H.A., Furtado R.N. (2015). Effectiveness of triamcinolone hexacetonide intraarticular injection in interphalangeal joints: a 12-week randomized controlled trial in patients with hand osteoarthritis. J. Rheumatol..

[12] Najm A., Alunno A., Gwinnutt J.M., Weill C., Berenbaum F. (2021). Efficacy of intra-articular corticosteroid injections in knee osteoarthritis: a systematic review and meta-analysis of randomized controlled trials. Joint Bone Spine.

[13] Tveter A.T., Blanck T., Nyheim S., Maarnes M., Christensen B., Pedersen S.J. (2022). OP0122-HPR Development of a smartphone application for treatment of hand osteoarthritis – Happy Hands (abstract). Ann. Rheum. Dis..

[14] Aebischer B., Elsig S., Taeymans J. (2016). Effectiveness of physical and occupational therapy on pain, function and quality of life in patients with trapeziometacarpal osteoarthritis - a systematic review and meta-analysis. Hand Ther..

[15] Ahern M., Skyllas J., Wajon A., Hush J. (2018). The effectiveness of physical therapies for patients with base of thumb osteoarthritis: systematic review and meta-analysis. Musculoskelet Sci Pract.

[16] Tveter A.T., Østerås N., Nossum R., Eide R.E.M., Klokkeide Å., Matre K.H. (2022). Short-term effects of occupational therapy on hand function and pain in patients with carpometacarpal osteoarthritis: secondary analyses from a randomized controlled trial. Arthritis Care Res..

[17] van Riel P.L.C.M., Scott D.L. (2004).

[18] Altman R., Alarcon G., Appelrouth D., Bloch D., Borenstein D., Brandt K. (1990). The American College of Rheumatology criteria for the classification and reporting of osteoarthritis of the hand. Arthritis Rheum..

[19] Haugen I.K., Felson D.T., Abhishek A., Berenbaum F., Bierma-Zeinstra S., Dziedzic K.S. (2023). EULAR classification criteria for hand osteoarthritis. Ann. Rheum. Dis..

[20] Kroon F.P.B., Damman W., Liu R., Bijsterbosch J., Meulenbelt I., van der Heijde D., Kloppenburg M. (2018). Validity, reliability, responsiveness and feasibility of four hand mobility measures in hand osteoarthritis. Rheumatology.

[21] American Academy of Orthopaedic Surgeons (1965).

[22] Schmidt R.T., Toews J.V. (1970). Grip strength as measured by the Jamar dynamometer. Arch. Phys. Med. Rehabil..

[23] Kellgren J.H., Lawrence J.S. (1957). Radiological assessment of osteo-arthrosis. Ann. Rheum. Dis..

[24] Altman R.D., Hochberg M., Murphy W.A., Wolfe F., Lequesne M. (1995). Atlas of individual radiographic features in osteoarthritis. Osteoarthritis Cartilage.

[25] Altman R.D., Gold G.E. (2007). Atlas of individual radiographic features in osteoarthritis, revised. Osteoarthritis Cartilage.

[26] Keen H.I., Lavie F., Wakefield R.J., D'Agostino M.A., Hammer H.B., Hensor E. (2008). The development of a preliminary ultrasonographic scoring system for features of hand osteoarthritis. Ann. Rheum. Dis..

[27] Hammer H.B., Bolton-King P., Bakkeheim V., Berg T.H., Sundt E., Kongtorp A.K., Haavardsholm E.A. (2011). Examination of intra and interrater reliability with a new ultrasonographic reference atlas for scoring of synovitis in patients with rheumatoid arthritis. Ann. Rheum. Dis..

[28] Mathiessen A., Haugen I.K., Slatkowsky-Christensen B., Boyesen P., Kvien T.K., Hammer H.B. (2013). Ultrasonographic assessment of osteophytes in 127 patients with hand osteoarthritis: exploring reliability and associations with MRI, radiographs and clinical joint findings. Ann. Rheum. Dis..

[29] Gløersen M., Hermann-Eriksen M., Hove Å., Kjeken I., Mathiessen A., Hammer H.B., Haugen I.K. (2024). Lessons learned from the PICASSO trial regarding ultrasound examinations of osteoarthritic hand joints performed by occupational therapists. Ultraschall der Med..

[30] Kroon F.P.B., Conaghan P.G., Foltz V., Gandjbakhch F., Peterfy C., Eshed I. (2017). Development and reliability of the OMERACT thumb base osteoarthritis magnetic resonance imaging scoring system. J. Rheumatol..

[31] Zhang W., Robertson J., Jones A.C., Dieppe P.A., Doherty M. (2008). The placebo effect and its determinants in osteoarthritis: meta-analysis of randomised controlled trials. Ann. Rheum. Dis..

